# Are early post‐discharge physician contacts associated with 30‐day psychiatric re‐hospitalisation? A nationwide claims data based retrospective cohort study in Austria free of immortal time bias

**DOI:** 10.1002/mpr.1983

**Published:** 2023-08-22

**Authors:** H. Katschnig, C. Straßmayr, F. Endel, M. Posch, I. Steiner

**Affiliations:** ^1^ Department of Psychiatry Medical University of Vienna Vienna Austria; ^2^ IMEHPS.research Vienna Austria; ^3^ Medical University of Vienna Center for Medical Data Science Institute of Medical Statistics Vienna Austria

**Keywords:** 30‐day readmission, ambulatory care, general practitioner, immortal time bias, psychiatric hospital department

## Abstract

**Objectives:**

Cost containment and quality of care considerations have increased research interest in the potential preventability of early re‐hospitalisations. Various registry‐based retrospective cohort studies on psychiatric re‐hospitalisation have focused on the role of early post‐discharge service contacts, but either did not consider their time‐dependent nature (‘immortal time bias’) or evaded the issue by analysing late re‐hospitalisations. The present study takes care of the immortal time bias in studying early psychiatric re‐hospitalisations.

**Methods:**

In a retrospective cohort study using nationwide electronic claims data in Austria, 10,689 adults discharged from acute psychiatric inpatient wards were followed up for 30 days. Cox regression analyses were performed with post‐discharge psychiatric and general practitioner contacts as time‐dependent covariates and time to first psychiatric re‐hospitalisation as outcome.

**Results:**

Post‐discharge ambulatory physician contacts were significantly associated with a decreased psychiatric re‐hospitalisation rate (hazard ratio 0.77 [95% CI 0.69; 0.87], *p* < 0.0001), with similarly strong contributions to this association by general practitioners and psychiatrists.

**Conclusions:**

Despite avoiding the immortal time bias and controlling for several confounders, we suggest to be cautious with a causal interpretation of the identified association, since potentially relevant confounders, such as disease severity, were unavailable in our claims data base.

## 1 INTRODUCTION

The 30‐day re‐hospitalisation rate is increasingly considered as an important health care quality indicator in medicine assuming that ‘bouncing back’ (Kind et al., 2007) after such a short time signals dysfunctional performance in the hospital, the aftercare or in the interplay between the two (Durbin et al., 2007; Griffith et al., 2022; Joynt & Jha, 2012). As far as early psychiatric re‐hospitalisation is concerned up to one in five patients discharged from a psychiatric inpatient episode is readmitted within 30 days in western industrialised countries (Donisi, Tedeschi, Salazzari, et al., 2016; Katschnig et al., 2019). Numerous studies have explored the role of baseline variables at the time of hospital discharge (such as age, gender, diagnosis or length of stay [LOS]) as potential predictors for psychiatric re‐hospitalisation for different post‐discharge time periods (Donisi, Tedeschi, Wahlbeck, et al., 2016; Tulloch et al., 2016). Yet, apart from the finding that the number of previous admissions increased the risk of re‐hospitalisation, results are inconclusive. Supposing that timely ambulatory follow‐up care might also matter, other studies have investigated the association of post‐discharge outpatient service use with re‐hospitalisation. Again, results are mixed, mainly due to differences in study setting, population, study design and methodology, as a systematic review has found (Sfetcu et al., 2017).

Apart from a few qualitative studies (Ådnanes et al., 2020; Sather et al., 2019), research exploring the role of continuity of care for psychiatric re‐hospitalisation is of two main kinds. The first type consists in mostly controlled small‐scale trials exploring the effect of specific transition interventions, which are mostly effective but are difficult to compare because of differences in the applied interventions (Vigod, Kurdyak, et al., 2013). The second type are observational studies with retrospective cohort study designs, using large claims databases reflecting the ‘real world’, which are becoming increasingly popular with the growing availability of electronic registry data. The methodological problem with the second type is that both the independent variable (outpatient contact) and the outcome (re‐hospitalisation) are time‐dependent and can only be studied with methods taking this time dependency into account, as, for example, Cox regression with time‐dependent covariates, in order to avoid the so called ‘time‐dependent bias’ or ‘immortal time bias’ (Dekkers & Groenwold, 2021; Shintani et al., 2009; Suissa, 2008; van Walraven et al., 2004, 2010).

While in other medical fields, for instance in pharmacoepidemiology (Acton et al., 2023; Suissa & Dell’Aniello, 2020) or in oncology (Heemskerk‐Gerritsen et al., 2019) awareness for time‐dependent biases is growing, this is not the case in the field of psychiatric re‐hospitalisation studies. Two studies examining the association between early post‐discharge contacts and the 30‐day psychiatric re‐hospitalisation rate, that is, the time period regarded increasingly as crucial as a quality indicator, did not—according to the description of their methods—consider the ‘immortal time bias’ and used standard multiple logistic or Cox regression with post‐discharge contacts as independent variable regarded as having been present at baseline (Cook et al., 2021; Huff, 2000).

A whole series of recent retrospective cohort studies on psychiatric re‐hospitalisation used large, often nationwide electronic databases for collecting information about outpatient contacts in the first weeks (mostly 30 days) after the index discharge from psychiatric hospitalisation, but examined the association of such early contacts only with re‐hospitalisations during the (mostly six) months *after* the critical early period (Beadles et al., 2015; Donisi, Tedeschi, Salazzari, et al., 2016; Hermer et al., 2022; Kurdyak et al., 2018; Lee et al., 2015; Marcus et al., 2017; Okumura et al., 2018). Findings were mixed. All these studies explicitly excluded patients with readmissions during the crucial first few post‐discharge weeks. They thereby avoided confrontation with the ‘immortal time bias’ at the expense of getting a very incomplete picture, as potential short‐term effects are not accounted for in the considered outcome measure. A recent follow‐up study on children and adolescents discharged from psychiatric inpatient care (Bardach et al., 2020) actually used ambulatory contacts in the first 30 post‐discharge days as time dependent covariate to study the association with re‐hospitalisation or emergency department visits in the 6 months after discharge, but did not report the 30‐day re‐hospitalisation rate.

Other than the above quoted papers, the present retrospective cohort study on psychiatric re‐hospitalisation, carried out with an extract from a nationwide claims database in Austria, addresses both the politically important 30‐day readmission rate and the avoidance of the immortal time bias by using Cox regression with post‐discharge ambulatory physician contacts as time‐dependent covariate. Whether such observational retrospective cohort studies, even if they avoid the immortal time bias, can lead to causal conclusions and are therefore useful for deriving measures to prevent ‘unnecessary re‐hospitalisation’ (Kim et al., 2021), is a different issue, which will be raised in the discussion section of this paper.

## 2 METHODS

### 2.1 Setting and data source

The study was carried out in Austria, a country with 9 million inhabitants, of whom nearly 100% have free access to medical care, because they are insured in a mandatory social health insurance (SHI) system, financed by a combination of payroll tax for outpatient care and general taxes for inpatient care. The basic structure of the ambulatory sector, both primary care and specialist outpatient care, consists of physicians working mostly in solo‐practices run as independent small businesses and paid by SHI institutions after claiming reimbursement for the provided services. It is noteworthy that in Austria no gatekeeping system exists, that is, patients can directly contact ambulatory specialist doctors (including psychiatrists) without a referral by a general practitioner (GP). Claims data for outpatient care are stored by several SHI institutions, but are not linked to hospital data, since hospitals are reimbursed by special hospital funds owned by nine provincial governments. Hospital data are collected by the provincial funds and stored in a specific federal database at the Ministry of Health. This fragmentation of responsibilities in the health care system (Bachner et al., 2018) prevents the comprehensive analysis of linked data for health care planning purposes.

For the present study it was possible to extract data from a specific research database, the GAP‐DRG (Endel et al., 2012) (see Supporting Information 1), established by a research consortium, linking pseudonymised nationwide service use data of primary care, specialist outpatient care and inpatient care in acute hospitals for the years 2006 and 2007. The basic structure of the healthcare system has not changed since then, but some restrictions apply. First, in order to improve interoperability between the different source databases, the number and granularity of variables in this research database are limited. Second, it does not contain data from private doctors who have no contract with a SHI institution and are paid directly by patients with some reimbursement for patients from SHI institutions (Stigler et al., 2013; for estimating the size of the private sector see Supporting Information 2). Third, some restrictions apply for psychiatric care: (a) the GAP‐DRG does not cover psychotherapy provided by private psychiatrists; (b) it does not contain data about the use of so‐called psychosocial services for psychiatric patients with persistent mental disorders living in the community (housing, day structure, counselling)—these services are very patchy across the country and are run by smaller or larger local NGOs, financed from local social budgets. Patients in need of such services are not entitled to use them (and must even occasionally co‐pay), since such community mental health services are not reimbursed by SHI. In sum, the variables used for the present study, do not represent the working of the whole mental health care system, but concern only that part of the psychiatric care system—acute psychiatric hospital units, GPs and outpatient psychiatrists—which nearly the whole population in Austria is entitled to use virtually free of charge.

### 2.2 Study population and variables

We first identified all adult patients (aged 18+ years) discharged with a main ICD‐10 diagnosis of F2–F6 (covering mainly schizophrenia, affective, anxiety and personality disorders) from any acute psychiatric inpatient unit in Austria in the year 2006, whereby only the first discharge in that year was selected. 21,839 patients fulfiled these criteria. In case of a psychiatric re‐hospitalisation within 30 days after the index discharge the number of days until this re‐hospitalisation was recorded. In a further step the number of days until the first physician contact (psychiatric and GP) was to be identified. In this step it turned out that seven of the then 13 major SHI funds had not recorded the exact date of the first post‐discharge physician contact, and the 10,969 patients belonging to these SHI funds had to be excluded. From the remaining cohort 176 patients were eliminated because of missing or unclear coding and five because they had died on the day of the hospital discharge (for details on the derivation of the study cohort see Supporting Information 3, Figure S1). The original cohort of all patients discharged in 2006 (*N* = 21,839) and the study cohort (*N* = 10,689) differ only slightly from each other in terms of the baseline variables (Table 1), which could be extracted from the GAP‐DRG, including female gender, age (equal to or higher than the median), LOS (equal to or higher than the median), a main diagnosis of a psychotic disorder (ICD‐10 F2 schizophrenia, F30 mania, F31 bipolar disorder), and physical comorbidity (at least one secondary physical ICD‐10 diagnosis; definition in Supporting Information 4).

**TABLE 1:** Comparison of baseline characteristics of the study cohort of the present study and of the preceding study (Katschnig et al., 2019).

Name of variable (names used in analyses)	Study cohort	Original cohort
	*N* = 10,689	*N* = 21,839
% women	59.0%	59.8%
Age median (Q1; Q3)	44 (33; 56) years	44 (34; 56) years
Length of stay median (Q1; Q3)	17 (8; 30) days	15 (8; 28) days
% psychotic disorder (ICD‐10: F2, F30, F31)	40.7%	36.2%
% physical comorbidity	32.6%	37.6%

### 2.3 Study design and data analysis

**FIGURE 1:**
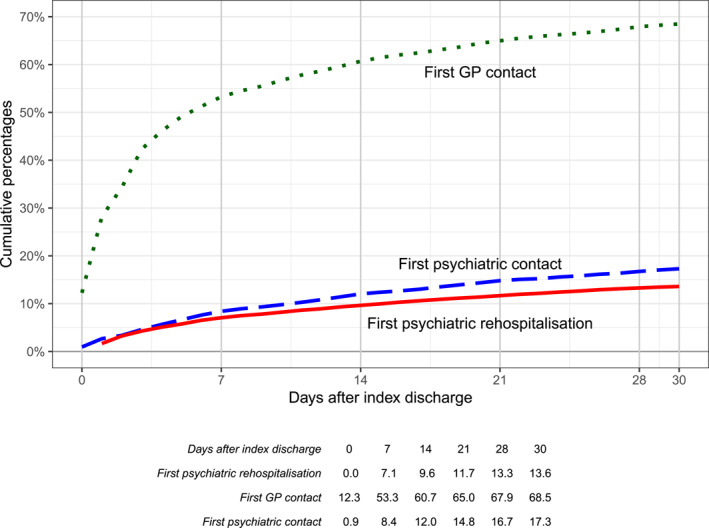
Time to first psychiatric re‐hospitalisation, first psychiatric contact and first general practitioner (GP) contact up to day 30 after the index discharge (*N* = 10,689). Cumulative percentages were calculated separately for each variable, that is, independently of their potential co‐occurrence.

In a first step, 30‐day post‐discharge frequencies for ‘first psychiatric re‐hospitalisation’, ‘first post‐discharge psychiatric contact’ and ‘first post‐discharge GP contact’ were calculated and cumulative frequencies plotted (independently of each other). In a second step, Cox regressions with time‐dependent covariates were performed with the time until first psychiatric re‐hospitalisation as dependent variable and with physician contact as ‘time‐dependent’ independent covariable to avoid the ‘immortal time bias’ (which would occur if the time‐dependent covariates were considered as having been present already at baseline). The statistical approach of using time‐dependent covariables accounts for the time patients were without or with physician contacts and models the risk of readmission in each state of the patient. Thus, in this model patients can switch the state (i.e., from no physician contact to physician contact) taking into account when the physician contact took place. This is done by splitting up the observation period of each patient into intervals without physician contact and intervals after a physician contact, respectively. The Cox regression model as opposed to the logistic regression model takes the individual time at risk into account by modelling the instantaneous hazard rate instead of an overall probability for readmission. Patients that died without a psychiatric re‐hospitalisation having occurred or patients that did not have any psychiatric re‐hospitalisation within the observation period of 30 days, were censored at the day of death or at day 30, respectively. In the first set of Cox regressions the first physician contact (regardless of whether it was a GP or a psychiatric contact) was used as the main independent variable. This variable is time‐dependent, that is, it is accounted for in the analyses that the group assignment (first psychiatric outpatient contact or first GP contact yes/no) is not fixed at baseline but may change during the observation period. For patients with such a physician contact, the time‐dependent variable was set to 0 until the day of the first contact, and to 1 from 1 day after the first contact to the day of psychiatric re‐hospitalisation or death or day 30, if no event occurred, respectively. For patients without any psychiatric outpatient or GP contact within 30 days, this variable was set to 0. In the second set of Cox regressions, a time‐dependent independent variable ‘type of physician contact’ was used in the model with the categories ‘no physician contact’, ‘GP contact as first physician contact’ and ‘psychiatric outpatient contact as first physician contact’ (with the equivalent coding as used in the first set of Cox regressions, but it was differentiated whether the first contact was a GP contact or a psychiatric outpatient contact by using a variable with three categories). All Cox regressions were performed both without and with the five dichotomous baseline variables as additional independent variables. Estimates for the hazard ratio with 95% confidence limits (HR [95% CI]) and the *p*‐value (null hypothesis: HR = 1) are reported for each independent variable. Because of the descriptive nature of the study, no adjustment for multiplicity was applied. The software R (R‐version 3.6.2, survival version 3.1–8) was used for the analyses (Core Team, 2019; Therneau, 2015; Therneau & Grambsch, 2000). Figure 1 was created with R/ggplot2.

## 3 RESULTS

### 3.1 Descriptive results

The descriptive statistics of the baseline variables of the study cohort are shown in Table 1. Three of five patients in the cohort were women and the median age was 44 years. Two out of five patients had a main diagnosis of a psychotic disorder at the index discharge (for frequencies of single main diagnoses see Supporting Information 5, Table S1) and nearly one in three patients had an additional physical comorbidity diagnosis. The median LOS was 17 days.

Frequencies of patients with 30‐day follow‐up events (independent of each other, also whether physician contacts occurred before or after a re‐hospitalisation) are shown in Table 2. About one in seven patients (13.6%) was re‐hospitalised to a psychiatric inpatient unit, 68.5% had at least one GP contact, and 17.3% at least one psychiatric contact. In comparison to the total cohort, re‐hospitalised patients had a higher proportion of a psychotic disorder, were younger and had a shorter LOS at the index discharge. The percentage of patients with a psychotic disorder at baseline was only marginally smaller in patients visiting a GP within 30 days after the index discharge (39.0%) than in those seeing a psychiatrist (41.9%). Patients visiting a GP were older and had a higher proportion of physical comorbidity than patients seeing a psychiatrist. Nearly three quarters of the cohort had a GP and/or a psychiatric contact. Considering the temporal sequence in this group, the overwhelming majority of first post‐discharge contacts was, with a tenfold difference, a contact with a GP and not with a psychiatrist (for this and other details about the chronological sequence of follow‐up events, see Supporting Information 6, Tables S2–S4).

**TABLE 2:** Patient groups with first psychiatric re‐hospitalisation, first psychiatric contact, first GP contact, death (up to post‐discharge day 30) and five baseline characteristics (for details on the sequence of events see Supporting Information 6).

Patient groups with different types of 30‐day follow‐up events	Group size		Baseline predictor variables				
	*N*	%	% female gender	% age =>44 years (median)	% LOS =>17 days (median)	% psychotic disorder	% physical comorbidity
Psychiatric re‐hospitalisation	1453	13.6	57.1	45.9	46.7	49.0	27.4
Psychiatric contact	1849	17.3	60.4	50.1	51.4	41.9	30.2
GP contact	7321	68.5	60.8	54.4	51.5	39.0	36.4
Death	47	0.4	44.7	70.2	44.7	31.9	53.2
Psychiatric and/or GP contact	7760	72.6	60.6	53.9	51.4	39.4	35.8
No GP & no psychiatric contact	2929	27.4	54.6	40.8	46.6	44.1	24.1
Total cohort	10,689	100.0	59.0	50.3	50.1	40.7	32.6

Abbreviations: GP, general practitioner; LOS, length of stay.

The time‐to‐event curves for follow‐up events (again independent of each other) are shown in Figure 1. A substantial proportion of events occurred already in the first week after the index discharge. Of all patients re‐hospitalised until day 30 (13.6%) more than every second (7.1%) was already re‐admitted during the first week after the index discharge. About the same holds true for the first psychiatric outpatient contact (17.3% at day 30, and already 8.4% at day 7). Of all patients with a first GP contact until day 30, more than three quarters (53.3% of 68.5%) had their first contact already in the first post‐discharge week.

### 3.2 Cox regression analyses with time‐dependent covariates

In the first set of Cox regressions a first GP and a first psychiatric outpatient contact were not distinguished—whichever occurred first was taken as the independent variable ‘first physician contact’, accounting for the time dependence of this variable, in order to avoid the ‘immortal time bias’. Table 3 shows that first physician contact is associated with a significant reduction of the 30‐day re‐hospitalisation rate, both in the unadjusted (Table 3a) and in the baseline covariable adjusted analysis (Table 3b). Except for female gender, all baseline covariables had a significant association with the re‐hospitalisation rate. Psychotic disorder at baseline was associated with a higher re‐hospitalisation rate, older age, longer LOS and physical co‐morbidity with lower rates.

**TABLE 3:** Cox regression with the first post‐discharge physician contact (GP or psychiatric, whichever was first) as time‐dependent independent variable and time to the first psychiatric re‐hospitalisation up to day 30 after the index discharge as dependent variable, without (a) and with (b) five baseline covariables (reference category for all independent variables = ‘no’).

Independent variables	(a) Unadjusted analysis		(b) Baseline covariable adjusted analysis	
	HR [95% CI]	*p*‐value	HR [95% CI]	*p*‐value
First physician contact (GP OR psychiatric)	0.74 [0.66; 0.82]	<0.0001	0.77 [0.69; 0.87]	<0.0001
Female gender			0.98 [0.89; 1.09]	0.76
Psychotic disorder			1.42 [1.28; 1.58]	<0.0001
Physical comorbidity			0.83 [0.74; 0.93]	0.0021
Age median or higher			0.89 [0.80; 0.99]	0.034
Length of stay median or longer			0.86 [0.77; 0.95]	0.0034

Abbreviations: GP, general practitioner; HR, hazard ratio.

In the second set of Cox regressions, it was differentiated, whether the first physician contact was a GP contact or a psychiatric outpatient contact. Both, first GP contact and first psychiatric outpatient contact, were associated with a significant reduction of the 30‐day re‐hospitalisation rate (first GP contact vs. no physician contact: 0.74 [0.66; 0.83], *p* < 0.0001, first psychiatric outpatient contact vs. no physician contact: 0.70 [0.54; 0.91], *p* = 0.008). The estimates of the hazard ratios were similar and the comparison of these two types of physician contacts did not reveal any statistically significant difference (HR [95% CI]: 0.94 [0.72; 1.23], *p* = 0.67). If adjusting for baseline covariables, similar results were obtained as in the unadjusted analysis (Table 4).

**TABLE 4:** Cox regression with type of first post‐discharge physician contact (categorised in first GP contact, first psychiatric outpatient contact and no physician contact) as time‐dependent independent variable and time to the first psychiatric re‐hospitalisation up to day 30 after the index discharge as dependent variable, without (a) and with (b) five baseline covariables (reference category for all independent variables = ‘no’).

Independent variables	Contrasts	(a) Unadjusted analysis		(b) Baseline covariable adjusted analysis	
		HR [95% CI]	*p*‐value	HR [95% CI]	*p*‐value
Type of first physician contact	First GP contact versus no physician contact (reference)	0.74 [0.66; 0.83]	<0.0001	0.78 [0.70; 0.88]	<0.0001
	First psychiatric outpatient contact versus no physician contact (reference)	0.70 [0.54; 0.91]	0.008	0.70 [0.54; 0.91]	0.009
	First psychiatric outpatient contact versus first GP contact (reference)	0.94 [0.72; 1.23]	0.67	0.90 [0.69; 1.17]	0.41
Female gender				0.98 [0.89; 1.09]	0.76
Psychotic disorder				1.43 [1.28; 1.58]	<0.0001
Physical comorbidity				0.83 [0.73; 0.93]	0.0019
Age median or higher				0.89 [0.80; 0.99]	0.033
Length of stay median or longer				0.85 [0.77; 0.95]	0.0033

Abbreviations: GP, general practitioner; HR, hazard ratio.

## 4 DISCUSSION

### 4.1 Patterns of 30‐day post‐discharge ambulatory physician contacts

Within 30 days after discharge from a psychiatric inpatient episode around one in six patients (17.3%) visited an office psychiatrist. This is more towards the lower end of the large range reported for visits to psychiatric outpatient services within 30 days post‐discharge in different health care systems, such as 9.1% for patients with schizophrenia, bipolar disorder and depression in state funded or operated mental health facilities in the USA (Hermer et al., 2022), 44% for schizophrenia in Canada (Kurdyak et al., 2018), 55.1% for any mental disorder in the national health service system in Italy (Donisi, Tedeschi, Salazzari, et al., 2016) and 85.1% for schizophrenia in Japan (Okumura et al., 2018). Such rates are not easily comparable, but it is of interest that in the Canadian study (Kurdyak et al., 2018) a comparison was performed between post‐discharge psychiatric and GP contacts, a comparison we were also able to perform. In our study more than two in three patients (68.5%) saw a GP within 30 days post‐discharge, in the Canadian study the GP visit rate was substantially lower (37%). The reverse holds true for psychiatric contacts—the rate of 44% in Canada is substantially higher than the 17.3% in Austria. This difference may be due to different availability of ambulatory services (Griffith et al., 2022) and other health system factors. A potential reason for these inverse patterns might also be the different diagnostic groups in the Canadian study (schizophrenia only) and in our study (all non‐organic psychiatric diagnoses), but this is improbable since in our study the proportion of patients with a baseline diagnosis of a psychotic disorder is nearly the same in patients visiting a GP (39%) as in those seeing a psychiatrist (41.9%). Given that in the 2 years covered by our database (2006 and 2007) the ratio of contract psychiatrists to contract GPs in Austria was one to thirty (for details see Supporting Information 2), psychiatrists were anyhow disproportionally more often contacted than GPs. Nine in 10 first physician contacts were with a GP and not with a psychiatrist (for details about the sequence of first contacts see Supporting Information 6, Table S4). In any case, in Austria GPs play a considerably larger quantitative role than office psychiatrists do in the first 30 days after discharge, and this despite the absence of a gatekeeping system in Austria, allowing to contact psychiatrists without a referral from a GP. The relevance of the primary care sector for early follow‐up contacts is underlined by the finding that three in four patients who contacted a GP up to day 30 did so already in the first post‐discharge week, while first contacts with psychiatrists rose less rapidly. One important reason for contacting a GP might be physical comorbidity, which is generally high in psychiatric patients (De Hert et al., 2011), and is higher in our study in patients who contacted a GP than in those contacting a psychiatrist. More general factors, such as stigma avoidance and less trust in psychiatry than in GPs may also play a role (Corrigan et al., 2014). Also, even though no ‘list system’ for primary care exists in Austria, many patients probably had ‘their GP’ already before the psychiatric inpatient stay, to whom they simply returned.

### 4.2 The association of early post‐discharge physician contacts with the 30‐day psychiatric re‐hospitalisation rate

About one in seven patients (13.6%) of the total cohort of 10,689 patients was re‐hospitalised within 30 days after the index discharge, which is within the range reported in the literature (Katschnig et al., 2019; OECD, 2013). A baseline diagnosis of a psychotic disorder strongly and significantly increased the 30‐day re‐hospitalisation rate, older age, physical comorbidity and longer LOS significantly reduced it. Gender played no role. Recent systematic reviews remain inconclusive about the effect of these baseline variables on the psychiatric re‐hospitalisation rate (Donisi, Tedeschi, Wahlbeck, et al., 2016; Šprah et al., 2017), presumably because of the largely differing methodologies used in different studies (Kim et al., 2021).

Concerning the association of time‐dependent early physician contacts with the 30‐day psychiatric re‐hospitalisation rate, we first did not distinguish between a first GP and a first psychiatric contact, that is, we used whichever came first after the index discharge as the independent variable ‘first physician contact’. After such a contact, the 30‐day rehospitalisation rate was strongly reduced. If first GP and first psychiatric contacts were considered separately, each of them was strongly associated with a reduced re‐hospitalisation rate. These results were obtained by using Cox regression with time‐dependent covariates, thereby avoiding the immortal time bias. This approach allowed to include also early re‐hospitalisations, which are a substantial fraction of total re‐hospitalisations, but are omitted in published registry‐based retrospective cohort studies on the association of early post‐discharge ambulatory contacts with psychiatric re‐hospitalisation (Beadles et al., 2015; Donisi, Tedeschi, Salazzari, et al., 2016; Hermer et al., 2022; Kurdyak et al., 2018; Lee et al., 2015; Marcus et al., 2017; Okumura et al., 2018).

We can conclude from our results that the first post‐discharge physician contact, regardless of whether with a GP or a psychiatrist, is significantly associated with a reduced psychiatric re‐hospitalisation rate in the first 30 days, and that the observed strength of the association is similar for GP contacts and psychiatric contacts. However, while our results seem to support the face validity of the concept that early continuity of care by psychiatrists and GPs is ‘causing’ a reduction of the psychiatric re‐hospitalisation rate, the statistical association identified is open to many different interpretations. The reason is that in retrospective observational studies confounding by unmeasured variables is always possible (Groenwold & Dekkers, 2020). Causal inference approaches, as the target trial approach (Hernán, 2021), could allow for stronger causal claims, but would rely on more detailed information on potential confounders, which is not available in our data set. While it was possible in our study to adjust for several potential confounders (gender, age, psychotic disorder, physical comorbidity, LOS), an association between early outpatient contacts and subsequent re‐hospitalisation rates might, for instance, have been mediated by the severity of the disorder, on which we had no information. A lower severity of the illness at baseline could have been related to both, a higher ability to access services and a reduced re‐hospitalisation rate (Cook et al., 2021). In an Australian study patients who received follow‐up care by the area mental health team within 7 days of discharge were more likely to be readmitted, and the authors suggest that patients who were selected by the mental health team for early contacts were already judged to be at greater risk of early readmission (Callaly et al., 2011). This caveat is also valid for the many recent retrospective registry‐based cohort studies quoted above, some of which implying that events in time period one (physician contacts) cause events (re‐hospitalisation) in a later time period, using already in the title terms like ‘impact’ (Kurdyak et al., 2018), ‘influence’ (Donisi, Tedeschi, Salazzari, et al., 2016) or speaking plainly of such early contacts as ‘lowering the risk of re‐hospitalisation’ (Lee et al., 2015).

### 4.3 Strengths and limitations

The decisive strength of our study is that we used Cox regression with time‐dependent covariates for assessing the association of post‐discharge ambulatory contacts with psychiatric re‐hospitalisation, thereby avoiding the immortal time bias, an approach not used so far in psychiatric re‐hospitalisation studies. Had we fallen prey to the immortal time bias by using the first post‐discharge physician contact not as time‐dependent covariate but as fixed baseline predictor, the association with re‐hospitalisation would have been, misleadingly, much stronger (HR 0.30 instead of correctly 0.74; see Supporting Information 7, Table S5). Another strength is the use of countrywide real‐world data and the large study population. This is an important advantage over randomised trials of transition interventions which always deal with selected groups of patients (Vigod, Kurdyak, et al., 2013). This implies that, in contrast to many other re‐hospitalisation studies, which focus on within‐hospital readmissions, our study is characterised by the system‐wide inclusion of health services, yielding a more realistic estimate of the total re‐hospitalisation rate (Vigod, Taylor, et al., 2013).

The main limitation concerns the use of data of an administrative registry with its predetermined set of variables with limited granularity and with information usually not collected for research purposes. The possibilities of accounting for confounding are therefore limited. For instance, in the database used in the present study no information was available on the legal status of a psychiatric hospital admission or on whether a re‐hospitalisation was planned or unplanned. Also, we cannot say anything about the kind and quality of ambulatory interventions. Severity of the disorder, a potentially important confounder, was only imperfectly measured by the diagnosis of a psychotic disorder. Also, because of missing calendar dates of physician contacts of some SHI institutions a large number of patients had to be excluded (which however did not substantially affect the sample characteristics). Concerning post‐discharge service covered in the ambulatory sector, visits to private physicians without a contract with a SHI fund are not included in the GAP‐DRG. The volume of care provided by these non‐contract physicians in the years 2006 and 2007 is not known but was rather limited at the time (see Supporting Information 2). Also, psychotherapy and counselling services provided by NGOs in the social sector are not included. Thus, the study deals exclusively with medical post‐discharge outpatient visits to contract physicians, GPs and psychiatrists, who are reimbursed by a SHI institution and provide their services free of charge to insured patients—and these comprise nearly 100% of the Austrian population. The transferability of the results of the present study to other countries is necessarily limited without considering the specific structure of other health care systems (Cetrano et al., 2020; Commonwealth Fund, 2017), including, for example, the number of available psychiatric hospital beds, payment mechanisms and related incentives for service providers, the mental health policy of a country and other background factors.

## 5 CONCLUSION

The strong association found between early post‐discharge physician contacts (both psychiatrists and GPs) and a reduced 30‐day psychiatric re‐hospitalisation rate is as such statistically valid, since the method used, that is, Cox regression with time‐dependent covariates, avoids the immortal time bias. However, while we adjusted the analysis for several potential confounders, confounding by unmeasured variables, such as severity of the disorder, cannot be ruled out given the retrospective observational study design based on registry data. Other than causal interpretations are therefore possible. What the use of linked registry data certainly allows, is to describe the longitudinal pattern of service utilisation, which can be useful information in itself for health care planners and politicians.

## AUTHOR CONTRIBUTIONS

**H. Katschnig**: Methodology; supervision; writing – original draft; writing – review & editing. **C. Straßmayr**: Investigation; writing – original draft; writing – review & editing. **F. Endel**: Data curation; visualization; writing – review & editing. **M. Posch**: Conceptualization; formal analysis; resources; validation; writing – review & editing. **I. Steiner**: Conceptualization; formal analysis; resources; validation; writing – review & editing.

## CONFLICT OF INTEREST STATEMENT

The authors declare no conflicts of interest.

## ETHICS STATEMENT

The study protocol was approved by the Ethical Committee of the Medical University of Vienna (EK Nr: 1302/2018).

## Supporting information

Supporting Information S1

## Data Availability

Research data are not shared.
