# Supplementary material for: Are early post‐discharge physician contacts associated with 30‐day psychiatric re‐hospitalisation? A nationwide claims data based retrospective cohort study in Austria free of immortal time bias
Source: Int J Methods Psychiatr Res. 2023 Aug 22;33(1):e1983. doi: 10.1002/mpr.1983 (PMC10804335; doi:10.1002/mpr.1983)
Supplement: Supplementary file 1 — Supporting Information S1 [file MPR-33-e1983-s001.docx]

**SUPPORTING INFORMATION**

**Contents**

[1. The GAP-DRG Database 2](#_Toc138327868)

[2. Office physicians in Austria 2](#_Toc138327869)

[a) Numbers of office psychiatrists and GPs with and without a contract with a Social Health Insurance Fund 2](#_Toc138327870)

[b) Ratio of availability of office psychiatrists with and without a contract with a Social Health Insurance Fund 2](#_Toc138327871)

[3. Derivation of the study cohort 3](#_Toc138327872)

[4. Definition of physical comorbidity 3](#_Toc138327873)

[5. Details for main ICD-10 F diagnoses of the study cohort 4](#_Toc138327874)

[6. Temporal sequence of follow-up events up to day 30 after the index discharge (psychiatric re- hospitalisation, first ambulatory psychiatric contact, first GP contact) 4](#_Toc138327875)

[7. Cox regression with post-discharge physician contacts used as baseline predictor, i.e., not taking into account the immortal time bias 5](#_Toc138327876)

1. **The GAP-DRG Database**

**Full name of the database in German (and English)**Datenbank für Grundlagenforschung für ambulante patientenbezogene Diagnosis Related Groups (General Approach for Patient-oriented Ambulant DRGs)
**Owner of the database**Main Association of Austrian Social Security Institutions
**Short description**The GAP-DRG is a pseudonymised electronic patient registry based on various claims databases for the acute inpatient, outpatient, pharmacy and other health sectors for the years 2006 and 2007. It includes service utilisation records of persons who are covered by the mandatory Austrian social health insurance system and for whom financial payment for health care utilisation was claimed. It covers nearly 100% of the Austrian population. Visits to privately paid physicians, emergency visits to acute care hospitals, and admissions to and discharges from military, prison, and forensic hospitals are not included.
**Reference**
Endel F, Endel G, Pfeffer N (2012) PRM34 Routine Data in HTA: Record Linkage in Austrias GAP-DRG Database. *Value in Health* **15**, A466.
Since 2019 the original GAP-DRG is not accessible anymore.

1. **Office physicians in Austria**
2. Numbers of office psychiatrists and GPs with and without a contract with a Social Health Insurance Fund

On 31 December 2006 (the midpoint of the years 2006 and 2007 which the GAP-DRG database covers) 3,943 GPs with a contract with SHI funds and 132 contract psychiatrists (excluding child psychiatrists) were practicing in Austria, corresponding to a ratio of roughly 30 GPs to one psychiatrist. Source of information: Mail from the Statistical Department of the Austrian Medical Chamber (Österreichische Ärztekammer) on 22 June 2021.

1. Ratio of availability of office psychiatrists with and without a contract with a Social Health Insurance Fund

No figures are available for the study years 2006 and 2007. A recent mapping project calculated that in 2017 in terms of opening hours of surgeries the availability of office psychiatrists/neurologists (still counted as one specialty for official statistics) without a contract with a Social Health Insurance Fund was around 10% of that of office psychiatrists/neurologists with such a contract. <http://www.dexhelpp.at/de/versorgungsatlas/task-2/ergebnisse/vergleich-1> (accessed 23-08-2022)

1. **Derivation of the study cohort**

**Supplementary figure 1: Derivation of the study cohort**

10,694

21,839

10,969 patients excluded
because their SHI fund did not register the exact date of the first post-discharge physician contact, thereof (number of excluded patients in brackets) 5 regional funds of the federal states of Burgenland (267), Upper Austria (4,400), Salzburg (1,527), Carinthia (1,479) and Tyrol (2,082), 2 country-wide funds – for railway & miners (485), farmers (660) - and several minor company funds (69). Remaining SHI funds: 4 regional funds (8,882: Vienna 3,066; Lower Austria 2,585; Styria 2,683; Vorarlberg 548) and 2 country-wide funds (1,988: civil servants 1,258; self-employed 730).

Adult patients (18+ years) discharged from hospital in 2006 with a main diagnosis of ICD-10 F2-F6

176 patients excluded
because of missing or unclear coding of contact date (thereof 109 of the SHI fund for self-employed persons because of missing data for the first quarter of 2006)

5 patients excluded
because they had died on the day of the hospital discharge

Study cohort

10,870

10,689

The original cohort of all patients discharged in 2006 (N = 21.839) and the study cohort (N = 10.689) differ only slightly from each other in terms of five baseline variables (see Table 1 in main manuscript).

1. **Definition of physical comorbidity**

Patients with physical comorbidity were defined as having at least one additional ICD-10 physical diagnosis. The rationale for the selection of ICD-10 categories was to include only "real" physical comorbidity. Therefore, ICD-10 categories, which do not represent a physical status that can be considered as comorbid as well as diagnoses which do not necessarily refer to physical comorbidity in terms of physical disease/disorder were excluded (e.g., categories V01–X59 which are used for coding accidents).

PHY_COM = at least one physical comorbidity diagnosis defined as at least one of the following ICD-10 diagnoses was coded at the index discharge as an additional/secondary diagnosis: A or B or C or D or E or (G00-G26) or (G35-G99) or H or I or J or K or L or M or N or (O00-O29) or (O95-O99) or S or T or (X60-Y34) or (Y40-Y98).

1. **Details for main ICD-10 F diagnoses of the study cohort**

**Supplementary table 1: Percentages of a) patients with specific main F diagnoses in the study cohort b) patients with a first 30-day psychiatric re-hospitalisation, first psychiatric contact and first GP contact in each diagnostic group**

|  | Cohort | | Percent first 30-day  post-discharge events | | |
| --- | --- | --- | --- | --- | --- |
| Main ICD-10 diagnosis | N | Percent | Psychiatric  re-hospitali- sation | First psychiatric contact | First  GP contact |
| F2 Schizophrenia, etc. | 3,414 | 31.9 | 16.5 | 16.7 | 64.2 |
| F30-F31 Mania/Bipolar disorder | 938 | 8.8 | 15.9 | 21.8 | 70.8 |
| F32-F39 Depression | 3,561 | 33.3 | 11.0 | 19.4 | 75.8 |
| F4 Anxiety disorders, etc. | 1,926 | 18.0 | 10.7 | 13.8 | 65.0 |
| F5 Somatoform disorders | 126 | 1.2 | 19.1 | 5.6 | 64.3 |
| F6 Personality disorders | 724 | 6.8 | 16.7 | 15.5 | 60.1 |
| Total | 10,689 | 100.0 | 13.6 | 17.3 | 68.5 |

1. **Temporal sequence of follow-up events up to day 30 after the index discharge (psychiatric re-hospitalisation, first ambulatory psychiatric contact, first GP contact)**

**Supplementary table 2: “First psychiatric re-hospitalisation” and “first ambulatory psychiatric contact” within 30 days after the index discharge (N of cohort followed up = 10,689)**

| Events | N | Percent |
| --- | --- | --- |
| No psychiatric contact and no re-hospitalisation | 7,582 | 70.9% |
| Psychiatric contact and no psychiatric re-hospitalisation | 1,654 | 15.5% |
| Re-hospitalisation and no psychiatric contact | 1,258 | 11.8% |
| Both events (regardless of their sequence in time), thereof | 195 | 1.8% |
| first psychiatric contact before first re-hospitalisation 111 |  |  |
| first psychiatric contact on same day as first re-hospitalisation 10 |  |  |
| first psychiatric contact after first re-hospitalisation 74 |  |  |

**Supplementary table 3: “First psychiatric re-hospitalisation” and “first GP contact” within 30 days after the index discharge (N of cohort followed up = 10,689)**

| Events | N | Percent |
| --- | --- | --- |
| No GP contact and no re-hospitalisation | 2,801 | 26.2% |
| GP contact and no re-hospitalisation | 6,435 | 60.2% |
| Re-hospitalisation and no GP contact | 567 | 5.3% |
| Both events (regardless of their sequence in time), thereof | 886 | 8.3% |
| first GP contact before first re-hospitalisation 611 |  |  |
| first GP contact on same day as first re-hospitalisation 41 |  |  |
| first GP contact after first re-hospitalisation 234 |  |  |

**Supplementary table 4:** **“First ambulatory psychiatric contact” and “first GP contact” within 30 days after the index discharge (N of cohort followed up = 10,689)**

| **Events** | **Abs.** | **Percent** |
| --- | --- | --- |
| No event | 2,929 | 27.4% |
| First GP contact and no ambulatory psychiatric contact | 5,911 | 55.3% |
| First psychiatric contact and no GP contact | 439 | 4.1% |
| Both events, thereof  Psychiatric contact before GP contact 302  Psychiatric contact on same day as GP contact 92  Psychiatric contact after GP contact 1,016 | 1,410 | 13.2% |
| “Pure GP contact group” - GP contact only (5,911) and first GP contact before first psychiatric contact (1,016) | 6,927 | 64.8% |
| “Pure psychiatric contact group” - Psychiatric contact only (439) plus first psychiatric contact before first GP contact (302) | 741 | 6.9% |

1. **Cox regression with post-discharge physician contacts used as baseline predictor, i.e., not taking into account the immortal time bias**

**Supplementary table 5:** Cox regression with the first post-discharge physician contact (GP or psychiatric, whichever was first) as independent variable and time to the first psychiatric rehospitalisation up to day 30 after index discharge as dependent variable, without (a) and with (b) five baseline covariables (reference category for all independent variables = “no”). The independent variable “first post-discharge physician contact (GP or psychiatric, whichever was first)“ was assigned the value 1, if any physician contact occurred within the first 30 days and prior to readmission if applicable, and the value 0 otherwise, i.e. the variable was treated as a baseline variable.

|  | 1. **Unadjusted analysis** | | 1. **Baseline covariable adjusted analysis** | |
| --- | --- | --- | --- | --- |
| **Independent variables** | **HR [95% CI]** | **p-value** | **HR [95% CI]** | **p-value** |
| First physician contact (GP OR psychiatric) | 0.30 [0.27; 0.33] | <0.0001 | 0.31 [0.28; 0.34] | <0.0001 |
| Female gender |  |  | 1.02 [0.92; 1.14] | 0.68 |
| Psychotic disorder |  |  | 1.37 [1.24; 1.53] | <0.0001 |
| Physical comorbidity |  |  | 0.91 [0.81; 1.03] | 0.12 |
| Age median or higher |  |  | 0.98 [0.88; 1.09] | 0.67 |
| Length of stay median or longer |  |  | 0.88 [0.80; 0.98] | 0.020 |
